# Special Issue “Role of Immune Cells in Non-Infectious Inflammatory Diseases and Cancers” †

**DOI:** 10.3390/ijms27125603

**Published:** 2026-06-21

**Authors:** Evgeny E. Bezsonov

**Affiliations:** 1 Laboratory of Molecular Virology, Sechenov First Moscow State Medical University (Sechenov University), Moscow 119435, Russia; bezsonov_e_e@staff.sechenov.ru; 2 Department of Biology and General Genetics, Sechenov First Moscow State Medical University (Sechenov University), Moscow 105043, Russia

## 1. Introduction

Inflammation is at the heart of many different non-infectious diseases, including cancer. The roles of different immune cells are highly context-dependent. They drive chronic inflammation and tissue damage in the case of inflammatory and autoimmune diseases, and they can also be used to mediate immune surveillance and eliminate tumors in cancer patients [1]. Nevertheless, when the tumor microenvironment is subverted, identical immune cell lineages can also drive malignancy and aid in evading immune surveillance [2]. Consequently, deciphering the diversity, adaptive versatility, and functional polarization states of immune cells is critical for developing precision therapeutics and improving patient prognoses across both pathological contexts [3].

Different immune cell types and their functional heterogeneity contribute to inflammation in different diseases.

Innate immune cells such as macrophages, neutrophils, mast cells, dendritic cells, and natural killer cells are directly connected with inflammatory response. Macrophages exhibit a high degree of plasticity, enabling their polarization into either the M1 (pro-inflammatory, anti-tumor) or M2 (anti-inflammatory, pro-tumor) subtype. In autoimmunity, M1 macrophages exacerbate tissue destruction, yet they are instrumental to anti-tumor immunity. In contrast, M2 macrophages, known as tumor-associated macrophages (TAMs), foster tumor growth, angiogenesis, and immune suppression [1,4,5,6]. By releasing reactive oxygen species (ROS) and proteases, neutrophils not only participate in acute inflammation but can also facilitate tumor advancement. Mast cells serve as key modulators of inflammation and angiogenesis, with established functions in chronic inflammatory conditions as well as in tumor development. Dendritic cells (DCs) are critical antigen-presenting cells whose maturation status dictates whether they promote effector T-cell activation or enforce immune tolerance. In the context of cancer, DCs frequently display defective antigen presentation as a result of tumor-mediated immunosuppression [1,4,5,6]. While natural killer (NK) cells are capable of killing tumor and virally infected cells, their anti-tumor activity is often dampened by the tumor microenvironment. Conversely, in autoimmune settings, NK cells can actively contribute to tissue damage [1,4,5,6].

Another type of cells, adaptive immune cells, include T and B lymphocyte subsets. Among CD4+ T cells, several functional subsets have been identified: Th1 (pro-inflammatory, anti-tumor), Th2 (involved in humoral immunity), Th17 (pro-angiogenic and pro-inflammatory), and regulatory T cells (Tregs), which exert immunosuppressive effects. Tregs are of particular significance due to their capacity to inhibit anti-tumor immunity and promote neoplastic growth [6,7]. CD8+ T cells serve as the primary mediators of anti-tumor immunity through their cytotoxic function. B cells are responsible for antibody secretion and immune response modulation; their contributions to cancer and inflammation are multifaceted and vary according to the pathological context.

Innate lymphoid cells (ILCs) are tissue-resident regulators of immune homeostasis and inflammation at barrier surfaces. When dysregulated, ILC subsets can promote both chronic inflammatory diseases and tumor progression [8,9,10]. Myeloid-derived suppressor cells (MDSCs) inhibit T cell function in cancer, thereby enabling immune evasion. Conversely, in autoimmune diseases, they can paradoxically exacerbate inflammation [8,9,10]. Both inflammatory conditions and neoplastic diseases feature an immune landscape defined by a wide array of innate and adaptive leukocytes, whose functions are highly dependent on the local context. Their inherent heterogeneity—at both the phenotypic and functional levels—governs the balance between host protection and pathogenic injury, thereby emphasizing the importance of precise immunological characterization for understanding disease mechanisms and guiding therapeutic strategies [1,4,5,6].

Different molecular and cellular pathways participate in immune cell-mediated disease progression. Key cytokines such as IL-6, TNF-α, and IL-10 are pivotal in modulating immune responses, angiogenesis, and immunosuppression. IL-6 and TNF-α promote tumor growth and neovascularization by activating STAT3 and NF-κB signaling, while IL-10 augments the immunosuppressive functions of T cells [11,12,13,14]. NF-κB pathway is activated by inflammatory signals and controls genes governing proliferation, survival, and immune responses. When activated within immune cells, NF-κB promotes both tumor progression and chronic inflammation [15,16,17,18]. The JAK-STAT pathway—particularly STAT3—plays a critical role in reprogramming immune cells within the tumor microenvironment. Inhibitors targeting this pathway can restore immune homeostasis and reverse tumor-induced immunosuppression [15,16,17,18]. During chronic inflammation, immune cells produce reactive oxygen species (ROS) and reactive nitrogen species (RNS), which cause DNA damage, promote genomic instability, and trigger mutations—events that fuel both the onset and advancement of cancer [19,20,21]. Dynamic crosstalk between immune cells, stromal cells, and cancer stem cells plays a pivotal role in driving disease progression and conferring therapy resistance. Such interactions reshape the tumor microenvironment, with consequential effects on immune cell infiltration, angiogenic responses, and the capacity for immune evasion [4,22,23].

DAMPs (Damage-Associated Molecular Patterns) and PAMPs (Pathogen-Associated Molecular Patterns) serve as key molecular signals that engage pattern recognition receptors (PRRs) on immune cells, thereby regulating inflammation and immune responses [24,25].

**Figure 1:**
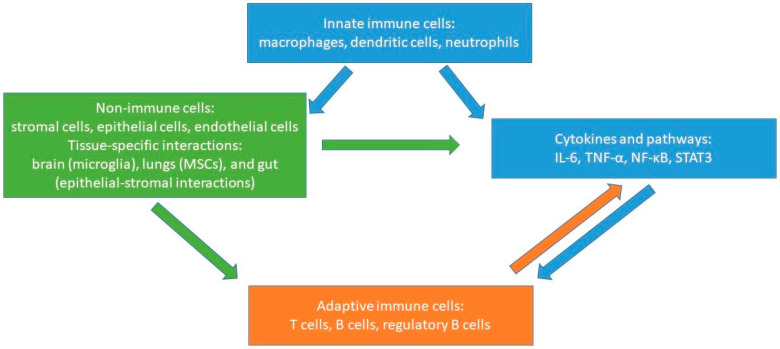
Interaction between immune cells in case of non-infectious diseases leading to chronic inflammation. Arrows of corresponding colors represent that the interaction is being initiated from the box with the corresponding color. MSCs—mesenchymal stem cells.

The interaction between immune cells in case of non-infectious diseases can be seen in Figure 1.

Macrophages, dendritic cells, and neutrophils—key constituents of the innate immune system—are the first to respond during inflammation. By producing cytokines and chemokines, they both initiate and sustain the inflammatory response, thereby recruiting adaptive immune effectors such as T and B cells [26,27]. Dendritic cells (DCs) engage in bidirectional crosstalk with epithelial cells to sustain immune homeostasis and initiate appropriate immune reactions. Dysregulation of this intercellular dialogue is a contributing factor to chronic inflammatory diseases, most notably within the pulmonary compartment [28]. Chronic inflammation is sustained largely by T and B lymphocytes. Within inflamed tissues, T cells release inflammatory cytokines, whereas B cells produce autoantibodies and function as antigen-presenting cells. This coordinated crosstalk generates a positive feedback mechanism that maintains long-term inflammation [29,30]. Non-immune cells—including stromal, epithelial, and endothelial cells—actively contribute to inflammation by secreting cytokines and chemokines. Through interactions with immune cells, these non-hematopoietic cells help to modulate the inflammatory response [31,32]. Stromal cells undergo a phenotypic switch toward a pathogenic state under chronic inflammatory conditions, thereby influencing the function of T and B lymphocytes and contributing to the chronicity of the disease [29,30]. Cytokines such as IL-6, TNF-α, and IL-1β are central mediators of inflammation. The IL-6 amplifier (IL-6 Amp), which engages the NF-κB and STAT3 signaling pathways, establishes a positive feedback loop between immune and non-immune cells that drives chronic inflammation [26].

Despite the large amount of available information on this topic, many blind spots remain, some of which will be addressed in this Special Issue. 

## 2. An Overview of Published Articles

Angel Yordanov’s article (contribution 1) is devoted to studying the expression of CD68 and CD47 in patients with differing tumor characteristics, histological variants of cervical cancer, and disease burden.

Dzhalilova et al. (contribution 2) focused on the morphology and molecular biology of hypoxia-tolerant and -susceptible colon tumors based on an assessment of colitis-associated colorectal cancer.

The third article, written by Pamonsupornwichit et al. (contribution 3), is devoted to the creation and investigation of a THP-1 cell line that is deficient in extracellular matrix metalloproteinase inducer (CD147), which is upregulated in different types of cancer, including T-cell acute lymphoblastic leukemia.

Jiménez-Gómez et al. (contribution 4) investigated B cells, T cells, and natural killer cells using multiparameter flow cytometry in silicosis caused by engineered stone patients and healthy controls.

The article by de Silva et al. (contribution 5) is devoted to identifying a positive correlation between T cell populations in peripheral blood and lung fluid of transplant recipients, with the CD8+Granzyme B+ effector T cell to regulatory T cell ratio emerging as a promising, minimally invasive blood-based biomarker for monitoring inflammation and chronic lung allograft dysfunction.

Khilwani et al. (contribution 6) studied IL-6 and IL-17, which promote lung cancer progression by polarizing macrophages toward an immunosuppressive M2 phenotype involving autophagy and inflammasome pathways. This information may help with the development of new therapeutic strategy for non-small cell lung cancer.

Four review articles were published in this Special Issue, addressing topics ranging from chronic inflammation, the role of immune cells in hepatic diseases and acute appendicitis to new approaches for coping with celiac disease.

The review by Miao et al. (contribution 7) is devoted to the current information on the role of CD4+T cells in nonalcoholic steatohepatitis (NASH) and hepatocellular carcinoma (HCC) progression. It discusses potential therapeutic approaches, including the targeting of CD4+T cells for the treatment of HCC and NASH.

Carvalho et al. (contribution 8) reviewed papers that explored the role of allergic response and Th2 reaction in the development of acute appendicitis.

The review by Liu et al. (contribution 9) is related to the role of high-temperature demand protein A2 and mitochondrial quality control in chronic inflammation.

The tenth article by Camarca et al. (contribution 10) is a review devoted to the effects of immunomodulating cytokines of celiac disease with a focus on Treg cells as potential therapeutic agents.

The Special Issue also includes a case report by Ioachim et al. (contribution 11), which describes a patient suffering from rare inflammatory disease—Riedel thyroiditis (RT). It also contains a literature review devoted to the variability of clinical manifestations of RT.

This Special Issue has covered the role of immune cells (T cells, B cells, NK cells, macrophages), cytokines (IL-6, IL-17), signaling molecules (CD68, CD47, CD147), and inflammatory mechanisms across a range of diseases, including cancers (cervical, colon, leukemia, liver, lung), inflammatory conditions (silicosis, appendicitis, chronic inflammation, celiac disease, Riedel thyroiditis), and transplantation-related complications.

## 3. Conclusions

Though there have been some achievements in the field, additional work is needed in order to uncover all the pathological details of sterile inflammation and the associated diseases. A complete understanding of the exact mechanisms governing immune cell plasticity and the dynamic modulation of immune checkpoints in the tumor microenvironment has yet to be achieved. Advancing techniques for long-term, non-invasive monitoring of adoptively transferred immune cells and establishing standardized manufacturing and quality control processes for cell-based therapies are essential topics for future investigation.

## Data Availability

Not applicable.
